# Adverse Childhood Experiences and Their Relation to Parenting Stress and Parenting Practices

**DOI:** 10.1007/s10597-018-0331-z

**Published:** 2018-09-07

**Authors:** Brittany C. L. Lange, Laura S. Callinan, Megan V. Smith

**Affiliations:** 1 Department of Social Policy and Intervention, University of Oxford, Barnett House, 32 Wellington Square, Oxford, OX1 2ER UK; 2 Department of Psychiatry, Yale School of Medicine, New Haven, CT USA; 3 Child Study Center, Yale School of Medicine, New Haven, CT USA; 4 Department of Chronic Disease Epidemiology, Yale School of Public Health, New Haven, CT USA

**Keywords:** Trauma, Adverse childhood experiences, Parenting stress, Parenting practices

## Abstract

The objective of this study was to understand the relationship between the early adverse childhood experiences (ACEs) of parents and their later parenting stress and practices. At the baseline visit of an 8-week course of cognitive behavioral therapy, parenting women completed the Parenting Stress Index-Short Form (PSI-SF) and the Positive Parenting Practices (PPP) scale. Linear regression procedures were used to assess the relationship between a parent’s own early experience of ACEs and current parenting stress and practices, including if there was a dose–response relationship. For the PSI-SF, significant dose–response relationships were observed between ACEs and the PSI Total Stress score (p < 0.05) and the difficult child subscale (p < 0.05). Additionally, a relationship was suggested with the parental distress subscale (p < 0.10). No significant relationships were found between ACEs and the parent–child dysfunctional interaction subscale of the PSI-SF or the PPP scale. Given the association observed between ACEs and parenting stress, it is important that future psychosocial interventions and policy initiatives preventing ACEs are developed.

## Introduction

Trauma represents a significant public health issue given its current prevalence in the United States (Kilpatrick et al. 2013). Typically, traumatic events are assessed through structured tools, including the Diagnostic and Statistical Manual of Mental Disorders (DSM) interview and the Traumatic Life Events Questionnaire (Peirce et al. 2009). Examples of traumatic events included on the DSM-5 interview for post-traumatic stress disorder (PTSD) include disasters, accidents, war exposure, experiencing or witnessing assault, and the death of, or threat of injury to, a close individual due to disasters, accidents or violence (Kilpatrick et al. 2013). While trauma has been assessed and defined in a number of ways, recent research using the DSM-5 found that 89.7% of individuals in the United States have experienced a traumatic event at some point in their lives (Kilpatrick et al. 2013).

Adverse childhood experiences (ACEs) are one conceptualization of trauma that have gained increasing attention in recent years. While the conceptualizations of trauma, such as the one found in the DSM-5, are fairly broad and include a number of experiences, such as war and disasters (Kilpatrick et al. 2013), typical scales used to assess ACEs do not encompass all of these experiences. Instead, typical assessments of ACEs include three general categories—childhood abuse, neglect, and household dysfunction (Centers for Disease Control and Prevention 2016). Additionally, ACEs are specific to a certain timeframe, as they only include experiences from when an individual was 18 years of age or younger (Centers for Disease Control and Prevention 2016). This is a critical component of the conceptualization of ACEs, as this early exposure to trauma, and the subsequent stress related to this repeated exposure, has been found to be associated with the disruption of a child’s developing brain (National Scientific Council on the Developing Child 2005/2014). For example, over time, this repeated exposure to stressful experiences (toxic stress), can alter stress responses and an individual’s ability to regulate themselves (Anacker et al. 2014; Loman and Gunnar 2010).

ACEs can result in a number of adverse outcomes for individuals. For example, ACEs have been associated with behavioral difficulties (Moore et al. 2014), adolescent pregnancy (Hillis et al. 2004), poorer educational outcomes and unemployment (Liu et al. 2013; Moore et al. 2014), substance use and abuse (Anda et al. 2006; Dube et al. 2003, 2006), poorer physical health outcomes (Anda et al. 2006; Chartier et al. 2010; Felitti et al. 1998), and poorer mental health outcomes or symptoms (Afifi et al. 2008; Anda et al. 2006; Chapman et al. 2004; Danese et al. 2009; Dube et al. 2001; Edwards et al. 2003; Whitfield et al. 2005), with a graded (dose–response) relationship being found between ACEs and a number of these outcomes (Afifi et al. 2008; Anda et al. 2006; Chapman et al. 2004; Chartier et al. 2010; Danese et al. 2009; Dube et al. 2001, 2003, 2006; Edwards et al. 2003; Felitti et al. 1998; Hillis et al. 2004; Moore et al. 2014; Whitfield et al. 2005). Further, research with low-income women has shown that specific ACEs, such as abuse and neglect, have been associated with reduced social support (Vranceanu et al. 2007).

Given the pernicious outcomes for both adults and children that result from trauma, several research studies have sought to examine how traumatic experiences may affect parenting practices. One study with low-income, parenting women found that “physical abuse was associated with increased hostile-intrusive behavior toward the infant,” while “sexual abuse was associated with decreased involvement with the infant” (Lyons-Ruth and Block 1996). Further, a number of studies have shown that outcomes associated with trauma, including mental illness, are associated with parenting behaviors, such as insecure parent–child attachment (bond) and decreased maternal sensitivity (responding to a child’s signals) (Downey and Coyne 1990; Lovejoy et al. 2000).

While specific types of trauma, such as physical abuse, sexual abuse, or neglect, have been studied in relation to future parenting practices (Bert et al. 2009; Hughes and Cossar 2016; Lyons-Ruth and Block 1996), there has been limited research specifically on the totality of ACEs in relation to parenting stress and practices (Chung et al. 2009; Steele et al. 2016). Additionally, though studies have focused on specific forms of childhood trauma and later parenting practices, the maximum age at which these acts of trauma can occur often vary by study. Thus, research focusing specifically on trauma a mother experienced before the age of 18 may be important. As such, this research aimed to (1) understand the relationship between a mother’s own experience of ACEs before the age of 18 and her current parenting stress and practices, as measured by several parenting scales; and (2) to determine if a dose–response relationship existed between a mother’s experience with ACEs and parenting stress and practices. Determining whether a dose–response relationship exists is especially important, as understanding the incremental nature of the effect of ACEs and other forms of trauma on parenting can allow us to better understand the mechanism whereby cumulative stress, trauma and adversity impact parenting and could potentially help to target interventions based on the moderating effect of the number of ACEs a mother has experienced.

## Methods

### The New Haven Mental health Outreach for MotherS (MOMS) Partnership

The MOMS Partnership is a collaboration between multiple community, state and academic agencies, that are dedicated to transforming “service delivery systems for mothers and children through community and neighborhood-based resources dedicated to wellness” (The New Haven MOMS Partnership 2018).

### Participants

Starting in 2012, the MOMS Partnership began administering needs assessments within the New Haven community. Women were recruited for needs assessments (described in the “Study Measures” section) by trained community mental health ambassadors (CMHAs) (Smith and Kruse-Austin 2015). CMHAs visited and worked in a number of locations within the community in an effort to recruit a representative group of parenting women (Smith et al. 2013). For further information on the needs assessment methods and the training of CMHAs, please see (Smith et al. 2013; Smith and Kruse-Austin 2015).

Needs assessments were used not only to determine the needs of the community, but to determine if parenting women were eligible for services offered by the MOMS Partnership. One service offered by the MOMS Partnership is a Stress Management course. As part of this course, parenting women participate in group cognitive behavioral therapy (CBT) over the course of 8 classes with other parenting women within the community. Inclusion criteria for the Stress Management course included that the woman: (1) served as a parent, primary caregiver or legal guardian of a child < 18 years of age; (2) scored a 16 or higher on the Center for Epidemiologic Studies Depression Scale (CES-D), which is a score indicative of depression; (3) was living in New Haven public housing, received Sect. 8 or were on the Rental Assistance Program.

**Fig. 1:**
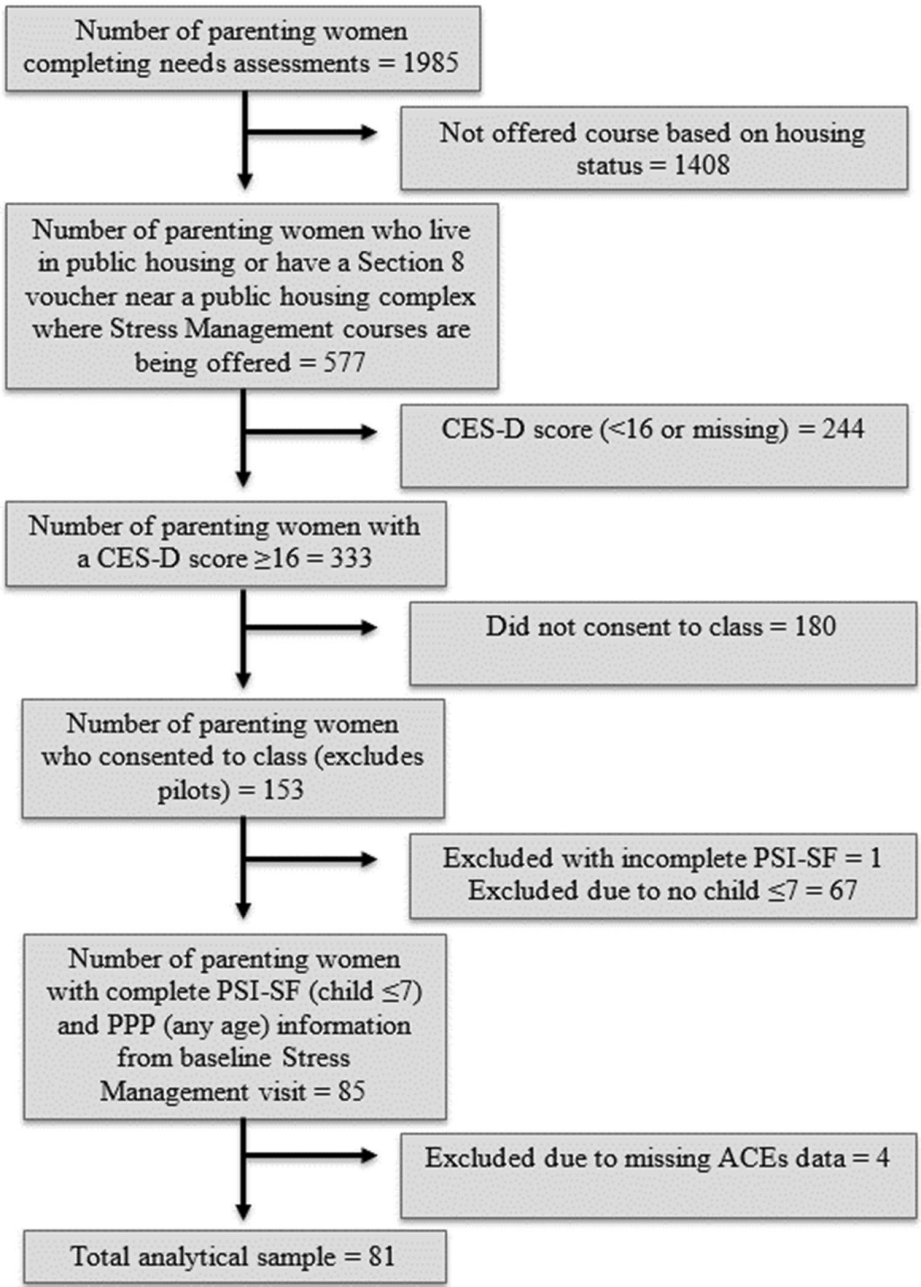
Consort diagram for stress management classes

Needs assessments were initially completed by 1985 women (Fig. 1). Of these women, 333 were eligible for Stress Management classes based on their CES-D scores and housing status. Ultimately, 153 parenting women enrolled in Stress Management courses. Of these, 68 were excluded from analysis for not having PSI-SF data or for having incomplete PSI-SF data, and 4 were excluded for not having ACEs data. Thus, 81 parenting women were included in the analytic sample for this study. Table 1 presents information on the demographic and clinical characteristics of these parenting women broken down by the presence or absence of ACEs.

**Table 1:** Demographic and clinical characteristics of parenting women (N = 81)

Characteristic	Total		No ACEs		ACEs	
	n	%	n	%	n	%
Age group (n = 74)						
18–24	9	12.16	4	16.67	5	10.00
25–34	30	40.54	7	29.17	23	46.00
35–44	24	32.43	9	37.50	15	30.00
45–54	9	12.16	4	16.67	5	10.00
55+	2	2.70	0	0.00	2	4.00
Race/ethnicity (n = 80)						
White, non-Hispanic	6	7.50	2	7.69	4	7.41
Black or African American, non-Hispanic	57	71.25	20	76.92	37	68.52
White, Hispanic	9	11.25	3	11.54	6	11.11
Black or African American, Hispanic	5	6.25	1	3.85	4	7.41
Other	3	3.75	0	0.00	3	5.56
Highest level of education achieved (n = 67)						
Elementary school (1–8 years)	2	2.99	1	4.35	1	2.27
High school (9–12 years)	36	53.73	13	56.52	23	52.27
College/vocational school or professional/graduate (13–20 years)	29	43.28	9	39.13	20	45.45
Are you currently employed? (n = 66)						
Yes, full time	4	6.06	3	13.64	1	2.27
Yes, part time	20	30.30	7	31.82	13	29.55
No	42	63.64	12	54.55	30	68.18
How would you rate your emotional health? (n = 78)						
Excellent	0	0.00	0	0.00	0	0.00
Good	15	19.23	7	26.92	8	15.38
Fair	43	55.13	15	57.69	28	53.85
Poor	14	17.95	2	7.69	12	23.08
Very poor	6	7.69	2	7.69	4	7.69
Have you received treatment for stress, sadness/depression or anxiety? (n = 80)						
Yes	27	33.75	7	25.93	20	37.74
No	53	66.25	20	74.07	33	62.26
Major depressive episode (n = 81)						
Yes	59	72.84	18	66.67	41	75.93
No	22	27.16	9	33.33	13	24.07
Post-traumatic stress disorder (n = 81)						
Yes	13	16.05	4	14.81	9	16.67
No	68	83.95	23	85.19	45	83.33
Panic disorder (n = 81)						
Yes	18	22.22	3	11.11	15	27.78
No	63	77.78	24	88.89	39	72.22
Generalized anxiety disorder (n = 81)						
Yes	45	55.56	13	48.15	32	59.26
No	36	44.44	14	51.85	22	40.74

### Procedures

#### Ethical Approval

Ethical approval for both the needs assessment and Stress Management course was obtained from the Yale University Institutional Review Board. Compensation for the needs assessment consisted of a gift card with a $10 value, while compensation for the Stress Management course was up to $370 for completing all assessments and classes.

#### Study Measures

##### Needs Assessment

The needs assessment tool is designed to capture information about the mother and her family, motherhood, basic needs, housing, and the physical and emotional health of mothers. Questions for the needs assessment were developed through an iterative process with a number of community stakeholders. Within the section regarding the physical and emotional health of mothers, parenting women were asked questions related to their own ACEs (Table 2), which were modified from the original ACEs questionnaire (Centers for Disease Control and Prevention 2016). The original ACEs questionnaire consists of questions on several adverse childhood experiences before the age of 18, as well as follow-up questions. For example, for child sexual abuse, the participant would be asked about the perpetrators of these acts, the numbers of acts that occurred, and examples of specific ACEs, such as “touching your sexual parts” and “intercourse…(oral, anal, or vaginal)” (Centers for Disease Control and Prevention 2016). However, for the purposes of the questionnaire for this study, women were asked eight yes/no questions about their experiences prior to the age of 18. Three of these questions were related to abuse and included emotional, physical, and sexual abuse. The remaining five questions concerned household dysfunction and included having an alcoholic or drug-user in the family, having an imprisoned family member, having a mentally ill family member, having a mother who was treated violently, or having both parents not present. A score for the ACEs was created by summing the results, with scores ranging from 0 to 8.

**Table 2:** Adverse childhood experiences (N = 81)

Characteristic	n	%
Experienced any ACE (n = 81)		
Yes	54	66.67
No	27	33.33
Number of ACEs (n = 81)		
0	27	33.33
1	14	17.28
2	13	16.05
3	11	13.58
4	3	3.70
5	9	11.11
6	1	1.23
7	2	2.47
8	1	1.23
ACEs by category (n = 81)		
Abuse		
Emotional abuse		
Yes	28	34.57
No	53	65.43
Physical abuse		
Yes	12	14.81
No	69	85.19
Sexual abuse		
Yes	29	35.80
No	52	64.20
Household dysfunction		
Alcoholic or drug-user		
Yes	22	27.16
No	59	72.84
Imprisoned family member		
Yes	22	27.16
No	59	72.84
Mentally ill family member		
Yes	14	17.28
No	67	82.72
Mother treated violently		
Yes	19	23.46
No	62	76.54
Both parents not present		
Yes	12	14.81
No	69	85.19

##### Parenting Stress Index-Short Form

The full Parenting Stress Index (PSI) consists of 101 questions that are “designed to measure the relative magnitude of stress in the parent–child system” (Abidin 2012). For the purposes of this study, the Parenting Stress Index-Short Form was utilized, and was administered at the baseline visit of the Stress Management course. The PSI-SF consists of 36 questions, which capture three domains—parental distress, parent–child dysfunctional interaction, and difficult child, with 12 questions asked for each of these domains (Abidin 2012). The Parental Distress subscale is designed to assess factors that may affect an individual’s parenting practices, such as limited social support, depression, or parental conflict with a partner (Abidin 2012). The Difficult Child subscale is designed to capture parenting challenges related to a child’s self-regulation or behavioral difficulties (Abidin 2012). Finally, the Parent–Child Dysfunctional Interaction subscale is designed to measure the “perception that the child does not meet…expectations” and is seen as a “negative element” in the life of a parent (Abidin 2012). The sum of all questions is used to capture the Total Stress score (Abidin 2012).

The PSI-SF has been validated and used in a number of contexts with parents (Abidin 2012; Ispa et al. 2004; Lecavalier et al. 2006; Smith et al. 2001). For analysis, the raw scores for the PSI Total Stress score and for each of the PSI-SF subscales were converted to corresponding percentiles, with the 90th–99th percentiles representing significant stress, the 85th–89th percentiles representing high stress, the 16th–84th percentiles representing normal stress, and the 0–15th percentiles representing below normal stress (Abidin 2012).

### Positive Parenting Practices

Positive Parenting Practices (PPP), which was also administered at the baseline visit of the Stress Management course, is a 6-item measure from the Chicago Youth Development Study, which measures how often parents have engaged in rewarding behaviors with their child during the previous 12 months (Dahlberg et al. 2005). Examples of rewarding behaviors include smiles, hugs, and special privileges (Dahlberg et al. 2005). Each item can be answered on a 1–5 scale (with 5 indicating almost always engaging in the listed behavior) (Dahlberg et al. 2005). The average score for the six items comprises the individual’s score on the assessment (Dahlberg et al. 2005).

### Data Analysis

Univariate analyses were run to determine the demographic and clinical characteristics of parenting women, their experiences with ACEs, parenting stress, and parenting practices. For the demographic and clinical characteristics analysis, results were separated into those who had not experienced an ACE, and those who had. Separate Fisher’s Exact tests were then performed utilizing the dichotomous variable for ACEs, and each of the demographic and clinical characteristics. These tests were performed to determine if these characteristics differed by whether an individual had experienced an ACE.

Individual linear regressions were conducted with each of the four PSI-SF scales and with the PPP scale to determine if a relationship, including a dose-relationship, existed between ACEs and these variables. No demographic characteristics were controlled for in these models. We hypothesized that controlling for any demographic characteristics would result in over-controlling given the significant homogeneity of our study population. To test this, we used Fisher’s Exact tests on ACEs and each of the demographic characteristics, as described above. These tests showed no significant associations between any demographic variables and ACEs, and thus, no demographic characteristics were controlled for in the model. Further, Fisher’s Exact tests were conducted with clinical characteristics, as described above. Given that these tests again showed no significant associations, and given that all women in the sample had symptoms indicative of depression as measured by scores of ≥ 16 on the CES-D, no clinical characteristics were controlled for.

## Results

### Demographic and Clinical Characteristics of Parenting Women

Table 1 presents information on the demographic and clinical characteristics of parenting women (N = 81). As Fisher’s Exact tests did not show statistically significant differences in the demographic characteristics by ACEs, characteristics are presented within the “Results” section for the entirety of the sample, regardless of ACE status. Overall, the majority of women in the study were between the ages of 25–44 (72.97%; n = 54). Additionally, most women reported being Black or African American (either Hispanic or non-Hispanic; 77.50%; n = 62). In terms of educational and occupational functioning, the majority of women had completed between 9 and 12 years of schooling (53.73%; n = 36) and reported being unemployed (63.64%; n = 42).

Many parenting women described challenges with emotional health, with 25.64% (n = 20) reporting that their mental health was poor or very poor. Additionally, 33.75% (n = 27) reported ever receiving treatment for emotional health challenges, including stress, sadness, or anxiety. Of the mental health conditions assessed, the most commonly reported were a major depressive episode (72.84%; n = 59) and generalized anxiety disorder (55.56%; n = 45).

### Experiences of ACEs and Parenting Practices

Overall, 66.67% (n = 54) of women reported experiencing at least one ACE (Table 2). Among these experiences, sexual abuse (35.80%; n = 29), emotional abuse (34.57%; n = 28), the imprisonment of a family member (27.16%; n = 22), and substance use of a family member (27.16%; n = 22) were the most common experiences reported by women.

On the PSI-SF, 17.29% (n = 14) had Total Stress scores indicative of high or significant stress (Table 3). Of the three subscales, the highest percentage of those with high or significant stress was observed on the Parent Distress scale (34.57%; n = 28) and the lowest percentage was observed on the Parent–Child Dysfunctional Interaction scale (9.87%; n = 8). On the PPP, the majority of women scored an average of 5 (88.89%; n = 72), which indicates almost always agreeing with all items on the scale (Dahlberg et al. 2005).

**Table 3:** Parenting stress and parenting practices (N = 81)

Scale	Total		No ACEs		ACEs	
	n	%	n	%	n	%
Parenting Stress Index-Short Form						
Total stress score						
Significant stress (90th–99th percentile)	10	12.35	3	11.11	7	12.96
High stress (85th–89th percentile)	4	4.94	1	3.70	3	5.56
Normal stress (16th–84th percentile)	62	76.54	21	77.78	41	75.93
Below normal stress (0–15th percentile)	5	6.17	2	7.41	3	5.56
Parental distress						
Significant stress (90th–99th percentile)	24	29.63	6	22.22	18	33.33
High stress (85th–89th percentile)	4	4.94	0	0.00	4	7.41
Normal stress (16th–84th percentile)	49	60.49	19	70.37	30	55.56
Below normal stress (0–15th percentile)	4	4.94	2	7.41	2	3.70
Parent–child dysfunctional interaction						
Significant stress (90th–99th percentile)	5	6.17	2	7.41	3	5.56
High stress (85th–89th percentile)	3	3.70	0	0.00	3	5.56
Normal stress (16th–84th percentile)	57	70.37	19	70.37	38	70.37
Below normal stress (0–15th percentile)	16	19.75	6	22.22	10	18.52
Difficult child						
Significant stress (90th–99th percentile)	14	17.28	2	7.41	12	22.22
High stress (85th–89th percentile)	3	3.70	0	0.00	3	5.56
Normal stress (16th–84th percentile)	51	62.96	19	70.37	32	59.26
Below normal stress (0–15th percentile)	13	16.05	6	22.22	7	12.96
Positive Parenting Practices (average score)						
5	72	88.89	26	96.30	46	85.19
4.33	6	7.41	0	0.00	6	11.11
3.67	2	2.47	1	3.70	1	1.85
2.33	1	1.23	0	0.00	1	1.85

### Relationship Between ACEs and Parenting Practices

**Fig. 2:**
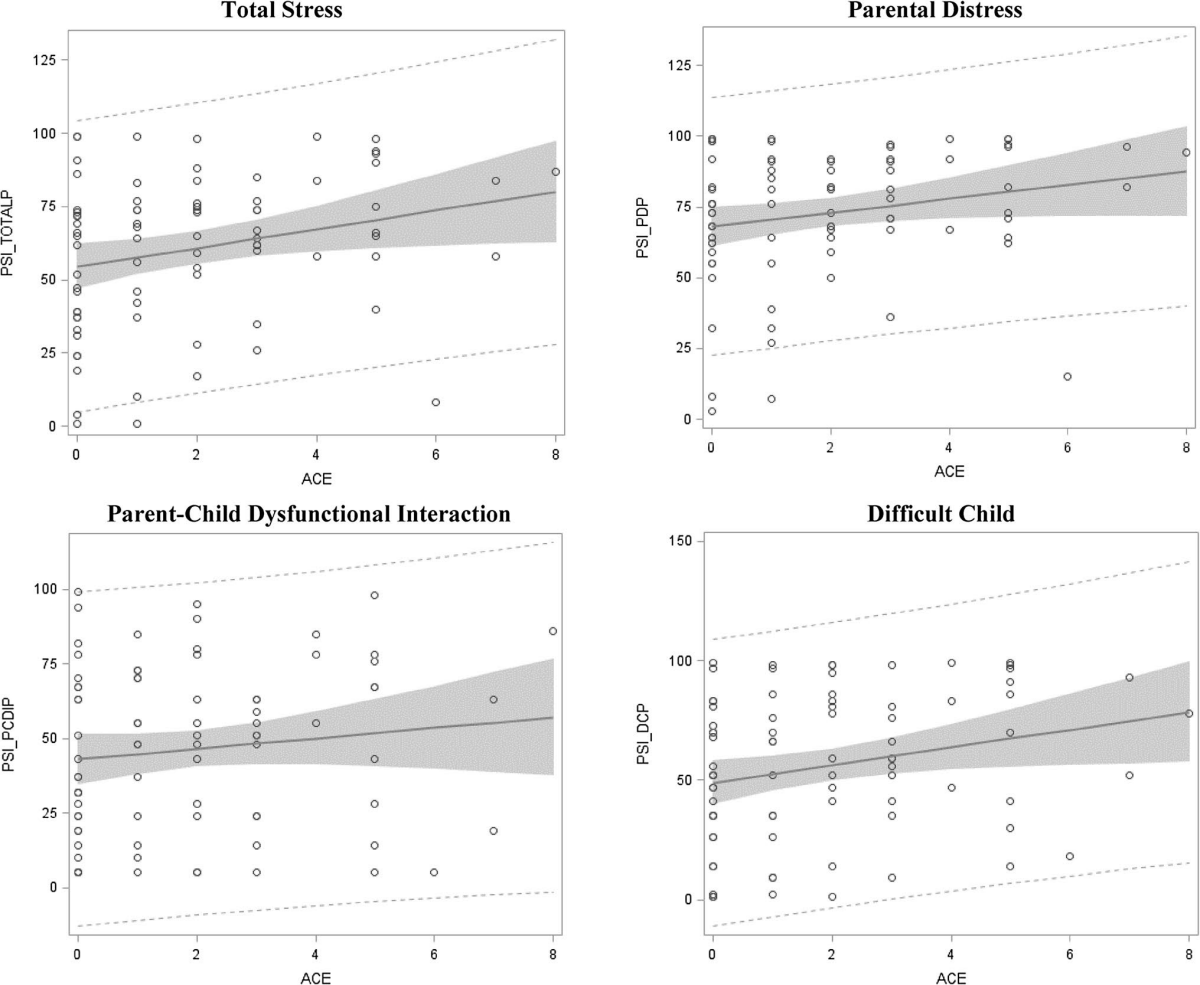
Dose–response relationship between ACEs and PSI-SF scales (N = 81)

Statistically significant relationships (p < 0.05) were found for both the Total Stress score and the Difficult Child subscale (Table 4; Fig. 2). Each additional ACE was found to be associated with a 3.19 increase in percentile for the Total Stress score and a 3.69 increase in percentile on the Difficult Child subscale. Additionally, a relationship suggestive of significance was found at p < 0.10 for the Parental Distress subscale, with each additional ACE being associated with a 2.45 increase in percentile on the subscale. ACEs were not found to be significantly associated with an increase in percentiles on the parent–child dysfunctional interaction subscale.

**Table 4:** Dose–response relationship between ACEs and parenting stress and parenting practices (N = 81)

Scale	Percentile or average score increase resulting from each additional ACE	P value
Parenting Stress Index-Short Form		
Total stress score	3.19	0.02*
Parental distress	2.45	0.05**
Parent–child dysfunctional interaction	1.76	0.26
Difficult child	3.69	0.03*
Positive Parenting Practices		
Average score	-0.001	0.95

*Statistically significant at p < 0.05

**Statistically significant at p < 0.10

No statistically significant relationships were found between ACEs and the average PPP score (Table 4).

## Discussion

Prior to this study, research had shown a relationship between specific traumatic events and parenting practices (Lyons-Ruth and Block 1996). However, limited research has been conducted specifically on the totality of a parent’s own early experience of ACEs in relation to a parent’s current parenting stress and practices (Chung et al. 2009; Steele et al. 2016). As such, this study aimed to understand this association, including whether a dose–response relationship exists, using measures of parenting stress (PSI-SF) and Positive Parenting Practices (PPP), in relation to a parent’s own ACEs.

Previous research has shown that ACEs have been associated with a number of negative long-term outcomes. This study was able to add to the existing literature by showing that more ACEs experienced early in life by a mother are positively associated with a mother’s current parenting stress, and that this association follows a dose–response relationship. Specifically, significant, dose–response relationships were observed at the p < 0.05 level for the PSI Total Stress score, where each additional ACE was associated with a 3.19 increase in percentile, and for the Difficult Child subscale, where each additional ACE was associated with a 3.69 increase in percentile. Additionally, at the p < 0.10 level of significance, each additional ACE was associated with a 2.45 increase in percentile on the Parental Distress subscale. However, no statistically significant relationship was observed on the Parent–Child Dysfunctional Interaction subscale of the PSI-SF. Further, no statistically significant relationship was observed for the PPP scale in relation to ACEs.

One potential mechanism for the increased parenting stress found in this study might be the dysregulation of the stress-response system caused by traumatic experiences as a child. Research has shown that early traumatic experiences can have an adverse effect on a number of important biological functions, and subsequent long-term outcomes, as they can cause “cumulative damage over time” and can embed “adversities during sensitive developmental periods” (Shonkoff et al. 2009). Further, recent research has shown that changes occur to the adult brain during the transition to parenthood (Kim et al. 2010, 2014), and these changes could be influenced by early childhood experiences, such as trauma.

For example, over time, biological mechanisms related to stress, including processes related to the hypothalamic-pituitary-adrenocortical (HPA) axis, are dysregulated, which prevent the body from “returning to homeostatic balance” (Shonkoff et al. 2012). Thus, individuals who have had these repeated exposures are likely to experience difficulty when faced with subsequent stressful experiences, as their stress systems have already been unduly burdened, and will not be able to as effectively regulate bodily processes related to stress. This may explain why parenting women in this study who experienced ACEs, and in particular, multiple ACEs, were shown to have increased levels of parenting stress, as measured by the PSI-SF.

Due to the increased levels of stress that parenting women who have experienced ACEs may have, and due to the subsequent dysregulation of the stress system, it is possible that the parenting styles of these women could be affected. Baumrind posits that there are four distinct parenting styles—authoritarian, authoritative, neglectful, and permissive (Baumrind 1991). One recent study has shown that “mothers who experience high levels of trauma symptoms are more likely to parent using authoritarian or permissive behaviors,” as measured by the Parenting Practices Questionnaire (which does not include neglectful parenting styles) (Leslie and Cook 2015). Further, a study looking at poor parenting practices, which includes factors such as neglect and aggression toward the child, found that maltreatment as a child was associated with poor parenting practices for mothers, and that childhood sexual abuse specifically was associated with aggressive parenting behaviors (Newcomb and Locke 2001). These findings are of critical importance, given that certain parenting behaviors captured within these parenting styles, such as neglect, are considered to be ACEs (Centers for Disease Control and Prevention 2016). Thus, it is possible for an inter-generational transmission of trauma to occur (Newcomb and Locke 2001).

As discussed, statistically significant associations on the Total Stress score, Parental Distress, and Difficult Child subscale with ACEs were found. The Parental Distress subscale is designed to assess factors, including stress, relationships, or mental health issues, that could affect an individual’s parenting practices (Abidin 2012). Research has shown that ACEs may make it difficult for individuals to cope with added stressors (American Academy of Pediatrics 2014), and as such, it is likely that increased ACE scores were associated with elevated percentiles on this scale, as parenting women may have had difficulty responding to additional parental stressors, such as parental conflict. Additionally, previous research has shown that early traumatic experiences are associated with reduced social support in adulthood (Vranceanu et al. 2007). Thus, parenting women may have seen elevated percentiles on the Parental Distress subscale due to more limited social networks, as more robust social networks, which could have helped buffer against stress, were not available. However, this study cannot confirm this association, as mediating factors were not measured.

The Difficult Child subscale is designed to capture difficulties that may occur for parents related to their child’s behavior (Abidin 2012). Recent research with parenting women has shown that mothers with mental health and substance use challenges were more likely to have children who experienced behavioral difficulties (Whitaker et al. 2006). Given that studies have shown that ACEs are associated with a number of adverse mental health outcomes (Afifi et al. 2008; Anda et al. 2006; Chapman et al. 2004; Danese et al. 2009; Dube et al. 2001; Edwards et al. 2003; Whitfield et al. 2005), it is possible that the increase in percentiles on the Difficult Child subscale, associated with the experience of each additional ACE, may be due to mental health outcomes related to the experience of trauma.

A significant association between ACEs and the parent–child dysfunctional interaction subscale was not found. This subscale is designed to measure the whether the child is seen as a negative part of a parent’s life (Abidin 2012). This finding could be due to the homogeneity observed in the sample on this measure, as only 8 individuals had scores indicating high or significant stress, with the majority of the sample having scores indicative of normal or below normal stress in this domain. Additionally, this result may be due to small sample size.

Finally, no significant association was found between the PPP scale and ACEs. This result is likely due to the significant homogeneity in results on this scale, as 72 of the 81 participants had an average score of 5, which is the highest possible score on the scale, and indicates that the mother almost always reports engaging in all behaviors (Dahlberg et al. 2005). It is possible that due to social desirability bias, women could have over-reported the rewarding behaviors described in the survey, perhaps believing that not endorsing some items, such as giving a “hug, pat on the back, or kiss” (Dahlberg et al. 2005), could be viewed unfavorably.

Given the relationship found between ACEs and parenting stress, it is important to develop both psychosocial and policy interventions to address these issues. Psychosocial research examining potential parenting interventions for adults will be especially critical, because research has shown that the negative effects of trauma can be lessened with early intervention (NGA Center for Best Practices, National Conference of State Legislatures, & Center on the Developing Child at Harvard University 2007), and as such, many interventions have been developed for younger populations (Wethington et al. 2008), while limited research has been completed related to adult populations.

Additionally, policies could be created, which seek to develop environments that are more sensitive and aware of the potential consequences of trauma. Policies creating more sensitive and aware school environments have been advocated for by the trauma-informed school movement (Cole et al. 2013), while policies creating more sensitive and aware community environments and service systems have been advocated for by the trauma-informed systems movement (Ko et al. 2008). Further, policies could be created to help aid adults who have experienced ACEs, such as policies that promote affordable access to appropriate mental health and parenting services.

Additionally, given that a dose response-relationship has been found in relation to ACEs and parenting stress within this study and in past studies related to ACEs and adverse outcomes (Afifi et al. 2008; Anda et al. 2006; Chapman et al. 2004; Chartier et al. 2010; Danese et al. 2009; Dube et al. 2001, 2003, 2006; Edwards et al. 2003; Felitti et al. 1998; Hillis et al. 2004; Moore et al. 2014; Whitfield et al. 2005), this research could be used to help inform genetic arguments. For example, Reiss et al. have posited a relationship between genes and the social environment, suggesting, among other mechanisms, that “genes can influence an individual’s response to environmental stress, genes may enhance an individual’s sensitivity to both favorable and adverse environments…” (Reiss et al. 2013). Thus, future studies should look not only at adverse outcomes of ACEs, but also at potential genetic influences.

## Strengths and Limitations

This study was able to address a critical gap in the literature on the effect that the totality of ACEs have on parenting stress and practices. This study benefited from the use of the PSI-SF, which is a validated measure that has been used in a number of studies with parents (Abidin 2012; Ispa et al. 2004; Lecavalier et al. 2006; Smith et al. 2001).

While the study was able to address this gap, there are a number of limitations that must be considered. Many of these limitations stem from the fact that this was a secondary analysis of data for a study not initially designed to assess the effect of ACEs on parenting. First, the sample size for this study was small. Given additional statistical power, it is possible that additional associations could have been identified. Second, this study focused on a homogenous population of low-income, parenting women living in public housing who scored highly on the CES-D. Thus, results may not be generalizable to other populations, including parenting men, those not living in public housing, those of other socio-economic statuses, and those not currently experiencing depressive symptoms.

Third, all measures of parenting relied on the self-report of mothers and thus single-source bias could exist. Additionally, these measures could be confounded by a mother’s own psychological status. Further, as with all parenting measures, social desirability bias may be present, which could lead to a lack of variability in responses, as was found with the PPP scale. It is possible that additional measures, such as the independent observation of parenting behaviors, could have helped triangulate the findings. While many studies investigating the associations between specific ACEs and parenting have relied on participant self-report, others have included participant observation to assess parenting behaviors (Bailey et al. 2012; Baumgardner 2007; Koren-Karie et al. 2008; Pasalich et al. 2016), and this could be an important avenue for future research. Additionally, it may be important to measure potential factors that may be affecting the parenting of mothers. Specifically, the behavior of children could affect the parenting stress a woman experiences. Thus, future studies may benefit from assessing the behavior of children through multiple sources. Further, potential mediators between ACEs and parenting, such as mental health, substance use, and financial issues, should be examined in future research.

Fourth, there are potential limitations related to the measurement of ACEs that must be considered. For example, questions related to ACEs, such as sexual abuse, are sensitive topics. As such, it is possible that women under-reported their experiences. Additionally, as described in the “Methods” section, ACEs were measured through eight yes/no questions. However, the full ACEs battery consists of additional questions, which may have more accurately assessed ACEs (Centers for Disease Control and Prevention 2016). Additionally, it is important to consider the distribution of ACEs within this sample. Specifically, the majority of the sample had ACE scores of three and under, with only 4 individuals having ACE scores of 6 and above. As such, there may be some uncertainty regarding the estimates in the higher range of the distribution. Finally, though there are benefits to measuring the totality of ACEs, some research has suggested that it is beneficial to instead examine the characteristics of specific adverse experiences (Dunn et al. 2018).

## Conclusion

Further research is needed to explore the relationship between adverse childhood experiences and parenting stress and practices. Future research should (1) utilize larger sample sizes; (2) utilize more diverse samples of individuals, including fathers; (3) examine parenting practices through other potential measures, including the Parent–Child Attachment—Rochester Youth Development Study scale, the Parental Involvement—Chicago Youth Development Study scale, and the Parental Supervision—Rochester Youth Development Study scale (Dahlberg et al. 2005); (4) examine potential mediators, such as social support; and (5) consider genetic influences. Additionally, future research should explore not only the relationship between ACEs and parenting stress and practices, but whether the parenting stress and practices resulting from these experiences affect child outcomes, including emotional, behavioral, and school-related outcomes.
